# Insidious Onset of Tetraparesis due to Cervical Epidural Abscess from *Enterococcus faecalis*

**DOI:** 10.1155/2013/513920

**Published:** 2013-03-20

**Authors:** Konstantinos Chr. Soultanis, Vasileios I. Sakellariou, Konstantinos A. Starantzis, Nikolaos A. Stavropoulos, Panayiotis J. Papagelopoulos

**Affiliations:** ^1^1st Department of Orthopaedic Surgery, University of Athens, Attikon University General Hospital, 1 Rimini Street, 12462 Chaidari, Greece; ^2^Department of Orthopaedic Surgery, Hospital for Special Surgery, 535 East 70th Street, New York, NY 10021, USA

## Abstract

We report a case of cervical epidural abscess from *Enterococcus faecalis*, which caused an insidious onset of tetraparesis. This 53-year-old female with a history of diabetes mellitus and chronic renal failure under hemodialysis presented with pain and progressive weakness of upper and lower extremities without fever. Although a recent MRI she did at the beginning of symptoms showed no significant pathologies, except for a cervical disc herniation and adjacent spinal degeneration, and stenosis that confused the diagnostic procedure, newer imaging with CT and MRI, which was performed due to progression of tetraparesis, revealed the formation of a cervical epidural abscess. Surgical drainage was done after a complete infection workup. The patient showed immediate neurological improvement after surgery. She received antibiotics intravenously for 3 weeks and orally for another 6 weeks. The patient was free from complications 24 months after surgery. A high index of suspicion is most important in making a rapid and correct diagnosis of spinal epidural abscess. The classic clinical triad (fever, local pain, and neurologic deficits) is not sensitive enough for early detection. Continuous clinical, laboratory, and imaging monitoring are of paramount importance. Early diagnosis and surgical intervention could optimize the final functional outcome.

## 1. Introduction

Cervical epidural abscess (CEA) is an uncommon pathological entity with potentially devastating neurological consequences [1, 2]. The incidence of spinal epidural abscesses (SEA) in general ranges between 0.2 and 2/10,000 hospital admissions but those located to the cervical spine only account for not more than 1% of all cases [1–3]. Although rare, they are a medical emergency because they are associated with significant morbidity and mortality if a delay in diagnosis and treatment occurs. 

Risk factors include diabetes mellitus, hemodialysis, spinal trauma, spinal surgery, bacteriemia, malignancy, and alcoholism [2, 4]. However, impaired immunological status is considered as the common background of these entities. The pathophysiology includes direct extension or hematogenous seeding in subjects with underlying predisposing factors that induce immunosuppression [2, 4]. Common pathogens include *Staphylococcus aureus* and epidermidis [5, 6]; however, due to the particular immunological status of these patients, other uncommon bacterial species can be cultured [7–10].

Although there is the so-called triad of epidural abscess (fever, local pain, and neurologic deficits) these are not sensitive enough to detect spinal abscesses early [2, 11, 12]. And despite advances in neuroimaging and surgical treatment, spinal and especially cervical epidural abscess remains a challenging problem. 

Herein, we present the case of a 53-year-old female with diabetes mellitus and chronic renal failure under hemodialysis, who insidiously developed neurological symptoms; these symptoms were initially attributed to a preexisting cervical degenerative disk disease (DDD) but turned to be an epidural abscess from *Enterococcus faecalis*. We try to highlight the nonspecific symptomatology as well as the importance of repeated clinical and imaging assessment in order to avoid disastrous or even lethal complications of false or late diagnosis and treatment [13, 14].

## 2. Case Report

**Figure 1:**
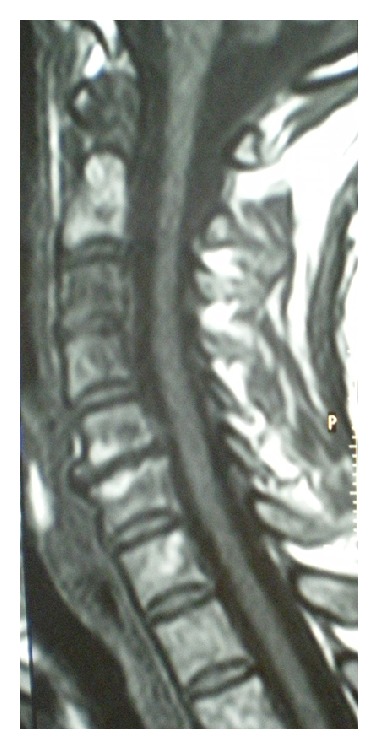
MRI sagittal view of the cervical spine showing a C5-C6 degenerative disc disease and generalized spondylosis of the entire cervical spine.

A 54-year-old woman presented to the emergencies with intermittent pain at the cervical spine. She referred no constitutional symptoms during the past period. However, she complained for upperextremities sensory deficits, during the past 3 weeks that were initially attributed to a known C5-C6 disk herniation (diagnosed through a recent cervical spine CT scan). For this reason she was advised by her attending physician to receive nonsteroid anti-inflammatory drugs and have a new cervical spine MRI (Figure 1). The MRI revealed herniated C5-C6 disc and associated spondylosis. She was referred to our department for further evaluation. During the past 12 hours though, the patient noted progressive weakness of both upper and lower extremities. 

From the patient's history, an end-stage renal failure under routine hemodialysis was noted that was a result of chronic noncontrolled type II diabetes mellitus. The patient also mentioned the history of a neglected L1 fracture that progressively caused significant discomfort and wedge deformity. This was causing progressive kyphosis and spinal canal stenosis and was successfully decompressed 6 months ago and stabilized posterior spinal instrumentation. She was nonsmoker and had no pathological drinking or drug habits.

On admission, neurological examination of the lower extremities showed increased deep tendon reflexes and muscle weakness documented as 4/5 on the right side and 3/5 on the left side. Marked hypoesthesia corresponding to the level of C3 was also noted. The tone of the anal sphincter was normal while the anal cutaneous reflex was absent. The temperature was repetitively measured and was within normal values. During the next hours, after admission to the hospital, the patient developed incomplete tetraparesis. 

**Figure 2:**
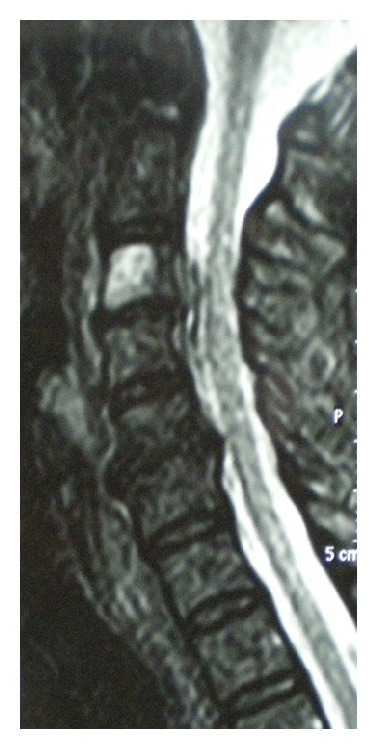
Preoperative MRI in sagittal plane demonstrating the spinal cord compression from a ventral C3-C4 epidural abscess.

The review of the available films of her recent MRI raised concerns regarding the C3-C4 region, and an urgent CT scan revealed C3-C4 spondylodiscitis. An urgent MRI in the view of the CT scan and laboratory findings demonstrated spinal cord compression due to a ventral C3-C4 epidural abscess (Figure 2).

Laboratory examination showed increased white blood cell count (13.920/*μ*L), abnormal erythrocyte sedimentation rate (ESR = 73 mm/hr), and C-reactive protein values (CRP = 61.8 mg/dL).

Further infectious screening workup consisting of a cardiac ultrasound and imaging of thoracic and lumbar spine was normal. The left internal jugular catheter (used for hemodialysis) was also inspected and blood samples were cultured. There were no obvious signs of infection around fistulas and catheters used for hemodialysis. Peripheral blood samples were also collected for culture. 

**Figure 3:**
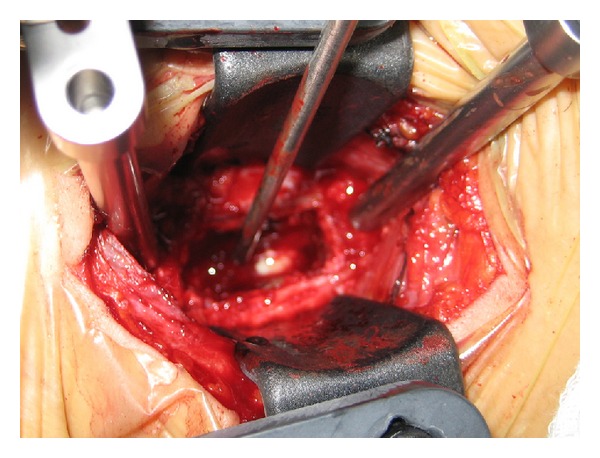
Intraoperative photo showing surgical debridement and drainage of the epidural abscess through an anterior cervical approach.

Due to severe and quick progression of neurological symptomatology, the patient was transferred immediately to the OR for surgical debridement and drainage of the epidural abscess through an anterior cervical approach (Figure 3). Partial resection of C4 vertebral body was performed in order to access the anterior epidural space. Meticulous drainage of the abscess and the surgical debridement took place and was followed by reconstruction of the anterior spinal column with a cage and plate. The immediate postoperative period was uneventful and the neurological symptoms were improved within 12 h after debridement and fully recovered by the 3rd postoperative day.

Leucocyte count was normalized on the fifth postoperative day. Erythrocyte sedimentation rate (ESR) and C-reactive protein (CRP) values showed a significant decrease after the 5th and 2nd postoperative days consecutively. Both intraoperative wound and preoperative blood cultures isolated *Enterococcus faecalis*, which was sensitive to linezolid, vancomycin, and teicoplanin. A course of 4-week intravenous vancomycin was followed by another 6-week period of parenteral (intramuscular) administration of teicoplanin. 

**Figure 4:**
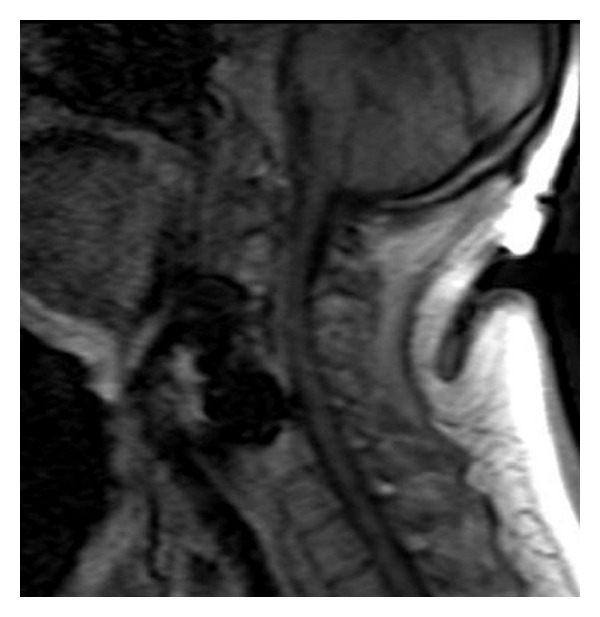
Postoperative MRI in sagittal plane confirming the drainage of the abscess and the subsequent release of the spinal cord pressure.

**Figure 5:**
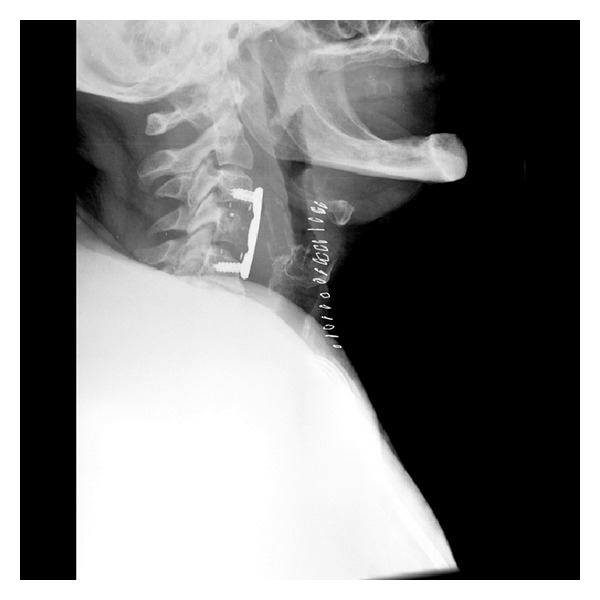
Postoperative lateral radiograph of the cervical spine showing a stable anterior C3–C5 stabilization using a cage and an anterior plate.

The postoperative MRI confirmed the drainage of the abscess and the subsequent release of the spinal cord pressure (Figure 4). The postoperative X-ray image showed a stable anterior C3–C5 fixation (Figure 5). The patient was discharged 23 days after surgery with a complete recovery of her neurological functions. 

She has now a followup of 24 months with no evidence of recurrence of the infection being off antibiotics for almost 21 months. 

## 3. Discussion

Cervical epidural abscess is a rather uncommon clinical entity. It represents almost the one percent of all spinal epidural abscesses, which have a reported prevalence of 0.2-1.2 cases per 10,000 admissions [1, 2]. Even though, the associated clinical importance overshadows the rarity of this clinical feature. The clinical presentation may be atypical. Confounding pathologies may mislead to or delay diagnosis with potential catastrophic consequences for the patient. In this paper, we have tried to highlight the importance of repeated clinical and imaging assessment in patients with suspected symptomatology and clinical background, even if initial findings are in favor of other diagnoses. 

Diabetes mellitus, hemodialysis, spinal trauma, and previous spinal surgery are acknowledged as the most important predisposing factors for the development of CEA [2, 4]. Our patient had all four: a history of insulin-dependent diabetes mellitus and associated chronic renal failure needing intermittent hemodialysis; she had also a history of recent lumbar fracture that was eventually reduced and stabilized through a posterior spinal approach. Other important factors include drug abuse, polymorbidity, AIDS, and other immunosuppressive pathologies. Morbid obesity is found to be an independent etiologic factor [2, 4].

With regards to the potential causative pathogens, *Staphylococcus aureus* is found to be the predominant organism isolated to almost 70% of reported cases [2, 4]. Rigamonti et al. in a large series of 75 spinal epidural abscesses found that except from *Staphylococcus aureus* (67%), other isolated microorganisms include methicillin-resistant *Staphylococcal* species in 15%, streptococcal infections in 7%, *Escherichia coli* in 5%, *Staphylococcus epidermidis* in 5% and *Pseudomonas aeruginosa* in 4% of the cases. *Klebsiella*, *Acinetobacter*, *Staphylococcus hominis* and *Mycobacterium tuberculosis* were each isolated in a rate of approximately 1% [4]. In the current literature, we could find no cervical epidural abscess caused by *Enterococcus* species. However, it is not unusual to culture relatively uncommon pathogens in patients with multiple comorbidities and/or immunological deficiencies [15–17].

Although clinical symptomatology can end up striking, spinal epidural abscesses are known to be entities in which “misdiagnosis is the rule, not the exception”. Initial clinical findings in CEA vary from slight cervical pain and fever to severe neurological disability. The clinical image has a tendency to progress insidiously. A “classic triad” of fever, spine pain, and neurologic deficits is recommended by several reports [2, 4]. Local pain is reported to be by far the most common presenting symptom (85%), but unfortunately this is not specific for the disease. Fever is present in a low percentage of patients. The use of antipyretic/analgesic medication before a scheduled visit to a physician may limit the significance of fever as a diagnostic criterion. However, repeated temperature measurements may be found useful and increase their sensitivity for diagnosis.

 Neurologic deficits are encountered in almost 40% of the patients. However, there is a wide spectrum of findings ranging from simple paresthesias to severe motor deficits with bowel and bladder incontinence. What is surprising though is the fact that neurologic symptoms are not always correlated to the radiographic image, and that the progression to complete tetraplegia could be fulminant [13, 14]. Usually, the early stages of CEA are characterized by solely local pain and tenderness over the spine. After these “quiescent” phases, an “acute” stage of the disease is entered, often heralded by a rise in temperature and acceleration of pain, ultimately resulting in weakness or paralysis. Davis et al., reviewing 63 cases with SEA, showed that this is a progressive disease, although the time course of symptoms is variable and unpredictable [18]. That is why an increased alertness and continuous monitoring of clinical, laboratory, and imaging alterations even within hours are needed in patients with the above-referred predisposing factors.

From the laboratory findings, the blood count usually shows an elevated white cells count and a left shift. Erythrocyte sedimentation rate (ESR) and C-reactive protein (CRP) are also increased above normal and are usually considered as more accurate laboratory findings for the followup of these cases [2, 4]. However, considering that these patients usually have multiple comorbidities and deficient immune responses, we understand that these findings are not specific for the disease. Positive blood cultures are found in only 60%, in comparison to wound cultures, which are positive in 98% of the cases. In our case, both blood and wound cultures were positive and revealed the same pathogen. 

MRI is considered the imaging modality of choice to identify a CEA. MR image characteristics are quite distinctive. The epidural mass may be iso- or hypointensive on T1-weighted imaging and is hyperintensive on T2-weighted imaging. Examination with gadolinium (Gd-DTPA) typically demonstrates linear enhancement surrounding non-enhancing purulent or necrotic matter [13, 14]. CT myelography is reserved only in cases in which MRI is not allowed [13, 14]. It shows clear compression of the thecal sac with or without complete block, which is highly suggestive of an epidural process. Plain radiographs may indicate only indirect signs and abnormalities, most frequently erosions of the vertebral body endplates adjacent to the disc space [13, 14]. An increased alertness should be shown regarding imaging findings, as they are not always correlating well with the severity of the neurological deficits. In our patient, although there were early clinical symptoms of pain and tenderness, the initial MRI did not reveal any pathology except for disc herniation and degenerative spine disease. Progression of neurological deficits urged for new imaging that revealed the recently formed CEA.

Meticulous surgical debridement and drainage of the abscess are generally recommended [19–21]. There are some reports of conservative treatment in selected cases of much-extended abscesses or significant surgical risks, but the outcome of conservative treatment is generally poor [22]. A prolonged course of intravenous antibiotics is usually recommended [19, 23]. These are commenced on an empirical basis and later tailored to the isolated pathogen. Although the exact duration of antibiotic therapy is not clearly defined, a minimum 3-week course of intravenous antibiotics is usually required [19, 23]. The total duration is related to the improvement of clinical image, radiographic findings and decrease in WBC count and inflammatory markers and ranges between 3 and 16 weeks [19, 23].

The outcome of SEA is usually determined by the timing of diagnosis and initiation of appropriate treatment [24]. Several parameters indicating poor outcome have been reported, including age greater than 60 years, spinal cord symptoms for longer than 72 hours, cervical SEA, extent of abscess more than 50%, overwhelming sepsis, low platelet count (<100 × 10^9^/L), and extremely high ESR (>110 mm/h) [25]. This may explain the good outcome and immediate clinical improvement after drainage of our patient despite her poor general physical condition. 

## 4. Conclusion

Cervical epidural abscess is a relatively uncommon but potentially devastating disease. Its clinical course might be insidious leading to significant delay in diagnosis and treatment. A high index of suspicion is of paramount importance. Patients with the “classic triad” of pain, fever, and neurologic deficits should be continuously monitored for clinical or radiographic changes. Surgical debridement and prolonged intravenous administration of antibiotics are the current standard of treatment. Uncommon pathogens may be isolated from the surgical wound in immune-compromised patients with multiple comorbidities. Early diagnosis and treatment are major factors for optimal outcome.
